# O031. Physiotherapy treatment in chronic tension-type headache: an ongoing study

**DOI:** 10.1186/1129-2377-16-S1-A175

**Published:** 2015-09-28

**Authors:** Antonella Monticco, Antonio Granato, Matteo Menegoni, Manuela Deodato, Jacopo Fantini, Roberto Marcovich

**Affiliations:** Physiotherapy Degree Course, University of Trieste, Trieste, Italy; Department of Medical, Surgical and Health Sciences, Neurologic Clinic, Headache Centre, University of Triese, Trieste, Italy

## Objectives

Aim of the study was to verify the efficacy of an individualized physiotherapy treatment based on a protocol assessment of cervical spine disorders in patients with chronic tension-type headache (cTTH).

## Methods

We enrolled patients with cTTH (ICHD3-beta criteria) attending the Headache Centre of Trieste who preferred not to take pharmacological treatment. Patients were prophylaxis free in the last 3 months and were asked to fill in an ad hoc diary in a three-month baseline period. The physiotherapy group (PhG) underwent a three-month combined protocol of postural advice, exercises and manual therapy. Intensity (NRS), frequency and duration of pain were analysed with GraphPad InStat 3.06. Cervical range of motion (CROM) was evaluated with a headgear provided with goniometer and spirit level. Neck pain was studied with the Neck Pain and Disability Scale I (NPDS) Questionnaire. A control group (CG) treated with amitriptyline for three months was compared with PhG.

## Results

We enrolled 8 PhG patients (5 M, 3 F; mean age, 58±16 years) and 8 CG patients (4 M, 4F; mean age, 49±21 years). Six of the 8 PhG patients improved, in 2 patients headache was eliminated, none worsened. All headache patterns were statistically reduced (pain intensity from NRS 5.7±1.6 to 2.2±2 [p = 0.007]; frequency from 26±5 to 15±13 days per month [p = 0.03]; duration of attacks from 14±7 to 5±7 hours [p = 0.01]). The patients who were headache-free had the most significant clinical improvement in the NPDS-I Questionnaire, while the 2 patients without improvement had null score. The NPDS score improved statistically after the treatment (p = 0.03). Most CROM improvement were Flexion 44% and Rotations (right: 75%; left: 48%). In the CG, duration (from 24 to 1 hour per crisis [p = 0.0001]) and frequency (from 23±3 to 7±6 days per month [p = 0.0005]) were significantly reduced, but no reduction in intensity (NRS from 3.25±0.7 to 2.5±0.9 [p = NS]) was noted.

## Conclusions

An individualized physiotherapy treatment based on combined exercises, manual therapy and postural advice, is efficient in improving headache, cervical motion and disability in cTTH. Individualized physiotherapy may represent an effective alternative option in treating patients with cTTH who prefer not to take prophylactic drugs.

Written informed consent to publish was obtained from the patient(s).

